# Occupational psychosocial stressors and ergonomic strain during pregnancy and sex-specific risk of childhood asthma

**DOI:** 10.1007/s00420-024-02107-6

**Published:** 2024-12-04

**Authors:** Mette Møller Dornfeldt, Sandra Søgaard Tøttenborg, Esben Meulengracht Flachs, Luise Mølenberg Begtrup, Ida Elisabeth Huitfeldt Madsen, Karin Sørig Hougaard, Camilla Sandal Sejbaek

**Affiliations:** 1 Department of Occupational and Environmental Medicine, Copenhagen University Hospital – Bispebjerg and Frederiksberg, Copenhagen, Denmark; 2 Department of Public Health, Faculty of Health Sciences, University of Copenhagen, Copenhagen, Denmark; 3 National Research Centre for the Working Environment, Copenhagen, Denmark; 4 National Institute of Public Health, University of Southern Denmark, Copenhagen, Denmark

**Keywords:** Occupation, Pregnancy, Psychosocial, Ergonomic, Asthma, Offspring

## Abstract

**Objectives:**

Previous studies have indicated that maternal occupational psychosocial stressors may affect the risk of asthma in the offspring, but their results are inconsistent. Maternal occupational ergonomic strain is associated with predictors of fetal lung development, including preterm birth and low birthweight; however, it is not known, whether ergonomic strain during pregnancy is a risk factor for asthma in offspring. The aim was to investigate maternal psychosocial stressors and ergonomic strain during pregnancy relative to the risk of offspring asthma.

**Methods:**

Live- and firstborn singletons (1996–2018) and their mothers were identified from Danish nationwide registers. Job code at time of conception was assigned to each mother and linked with exposure estimates from job exposure matrices (JEMs) of psychosocial stressors and ergonomic strain. Diagnoses of childhood asthma were retrieved from the Danish National Patient Register. Incidence rate ratios (IRR) of asthma were estimated using Poisson regression; adjusted for maternal asthma, age at conception, socioeconomic position, and body mass index, and calendar year.

**Results:**

Maternal employment in occupations with low decision authority (IRR: 1.08, 95% CI 1.00–1.16) and high ergonomic strain (IRR: 1.09, 95% CI 1.02–1.16) was associated with increased risk of asthma among male offspring. Largely similar, but less consistent, associations were observed among female offspring due to low decision authority.

**Conclusion:**

We found a minor increased risk of asthma among offspring whose mothers worked in an occupation with low decision authority or high ergonomic strain, most pronounced among male offspring.

**Supplementary Information:**

The online version contains supplementary material available at 10.1007/s00420-024-02107-6.

## Introduction

Asthma is the most common chronic childhood disease. It is characterized by chronic airway inflammation with symptoms such as wheezing and/or coughing (Ferrante and La Grutta 2018; Global Initiative for Asthma 2022; Innes Asher et al. 2020). The prevalence of self-reported asthma in Danish children and adolescents was 5% in 1986 and had increased to 12% in 2018 (Thomsen et al. 2004, 2018). The prevalence is marginally higher among females than males in a life-time perspective, but males tend to be diagnosed before puberty and females during and after puberty (Bacharier et al. 2008; Chowdhury et al. 2021; Skadhauge et al. 2005). The underlying biological explanations for this sex-difference is unclear (Bacharier et al. 2008), but may include sex-specific vulnerability to environmental exposures during fetal life (Chowdhury et al. 2021; Saif et al. 2014).

In Denmark, the prevalence of women working during reproductive age has been 70–80% over the past 15 years (Statistics Denmark 2022; The Ministry of Employment 2022), and most Danish women work during pregnancy. Since the beginning of this century, work environment conditions have somewhat changed. While experience of psychosocial stressors has become more common at work, fewer experience ergonomic strain (Van Der Noordt et al. 2019).

Previous studies examining the association between maternal occupational psychosocial stressors and offspring asthma have showed inconsistent associations (Larsen et al. 2014; Liu et al. 2019; Magnus et al. 2018; Pape et al. 2020). Prenatal exposure to psychosocial stressors and ergonomic strain has been suggested to influence immune development (Clapp 2003; Rakers et al. 2020; Sternfeld 1997; Wright 2008; Wright et al. 2010). Transfer of stress-induced maternal cortisol to the fetus across the placenta has traditionally been considered the primary mediator of prenatal stress (Rakers et al. 2020; Wright 2008). There is also some evidence that maternal stress may stimulate placental release of corticotropin-releasing hormone, also into the fetal circulation, where it may stimulate secretion of glucocorticoids from the fetal hypothalamic–pituitary–adrenal (HPA) axis. Elevated fetal cortisol levels may interfere with development of the immune system. However, elevated cortisol levels in the prenatal circulation may also change the set point of the negative feed-back systems of the human fetal HPA axis, also with consequences for immune function later in life (Rakers et al. 2020; Wright 2008). Findings from animal and human studies suggest that maternal stress skews immune imbalance towards T helper 2 cell predominance in the fetus which predisposes to asthma and allergic diseases (Veru et al. 2014; Wright et al. 2010). Today, several pathways are, however, thought to be involved in in utero programming of offspring immune function (Rakers et al. 2020; Wright 2008).

For ergonomic strain, two meta-analyses of maternal exposure to occupational ergonomic strain showed associations to preterm birth and low birthweight (Cai et al. 2020; Croteau 2020), which again is associated with impaired lung function and increased risk of offspring asthma (Dezateux et al. 2004; Liu et al. 2014; Xu et al. 2014; Zhang et al. 2018). The mechanism(s) underlying the association between maternal occupational ergonomic strain and the adverse birth outcomes is not fully elucidated. It is suggested that temporarily reduced uterine blood flow could reduce nutrient delivery to the fetus, which may impair fetal growth (Clapp 2003; Sternfeld 1997). Whether maternal occupational ergonomic strain in fact constitutes a risk factor for offspring asthma is however yet to be examined.

We hypothesize that maternal exposure to psychosocial stressors and ergonomic strain during pregnancy independently increases the risk of offspring asthma. The aim of this study was to investigate the association between maternal occupational psychosocial stressors on offspring asthma in a large, unselected sample with long follow-up, and, for the first time, investigate the association between occupational ergonomic strain during pregnancy and offspring asthma. To do so, we used data from an early version of the DOC*X-Generation cohort with data from nationwide Danish registers and exposures assessed by Job Exposure Matrices.

## Methods

### Study design

This study was conducted using an early version of the DOC*X-Generation (DOC*X-G); a population-based, nationwide birth cohort within the EXIMIOUS (Mapping the Exposure-Induced Immune Effects: Connecting the Exposome and the Immunome) project (EXIMIOUS 2024). The DOC*X-G is an extension of the Danish Occupational Cohort with eXposure data (DOC*X) (Flachs et al. 2019), including pregnancies of all the females in DOC*X and their offspring with information from the Fertility Database (Statistics Denmark). DOC*X is constructed from the Occupation and Industry Register (OIR) and other Danish population-based registers (Flachs et al. 2019), all available at Statistics Denmark. Information from the registers was linked using a pseudonymized version of the unique Danish personal identification number (Thygesen et al. 2011).

### Study population

**Fig. 1:**
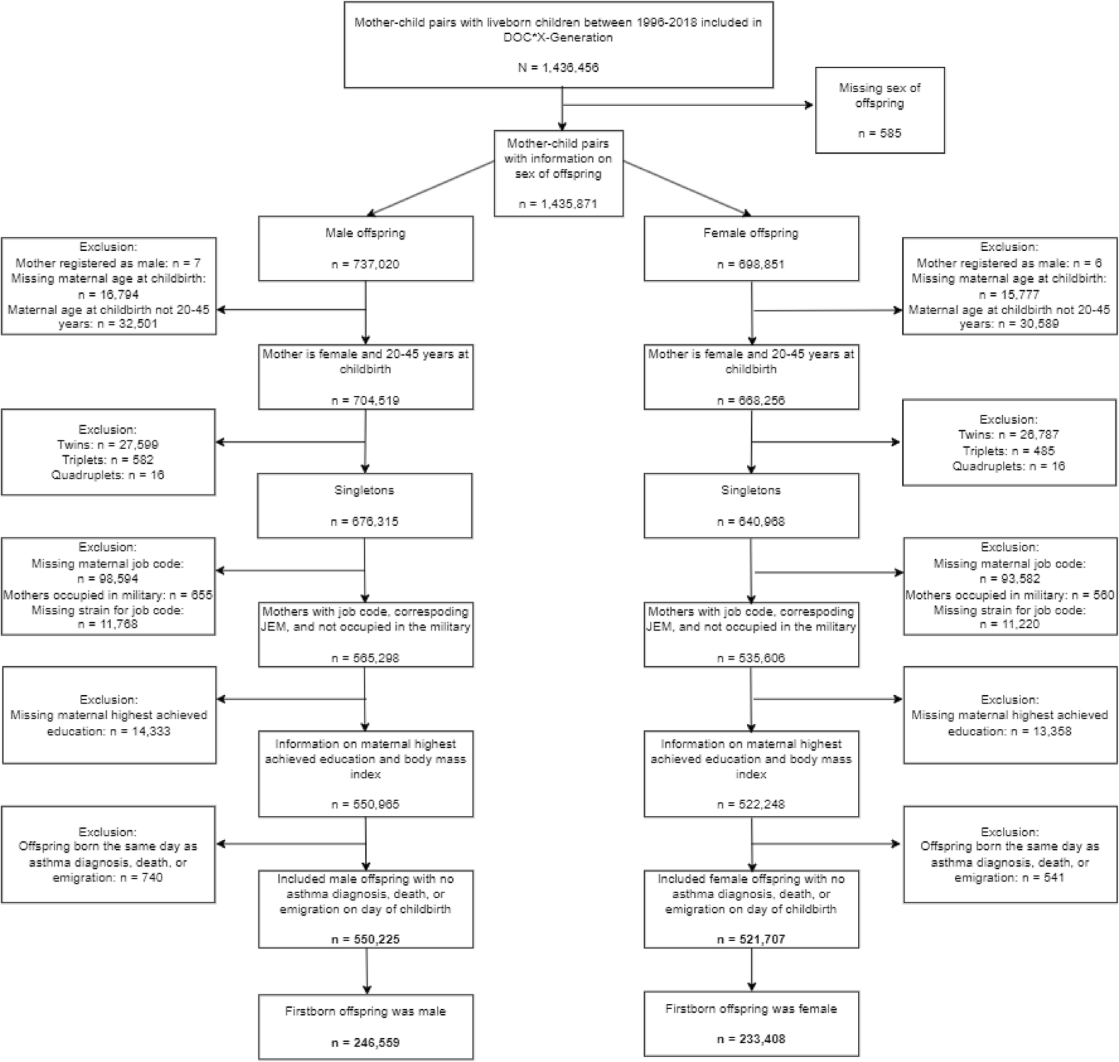
Flowchart of the study population in DOC*X-Generation

A total of 1,436,456 mother–child pairs with liveborn offspring born 1996–2018 were identified in DOC*X-G. Offspring with missing information on sex were excluded from the study population. Due to the hypothesis of sex-specific vulnerability, the study population was split into a male and a female offspring cohort. Mother–child pairs were excluded if mothers: were registered as males; were younger than 20 years or older than 45 years of age at conception; worked in the military (this job code is not specified into details of occupational tasks); lacked job code during pregnancy; lacked job exposure matrix (JEM)-measures for the psychosocial stressors and/or ergonomic strain for the job code; or lacked information on relevant confounders. Additionally, mother–child pairs were excluded if the offspring was not a singleton, or the day of childbirth was similar to the day of asthma diagnosis, death, or emigration for the offspring. Finally, the study population was restricted to the mothers’ first liveborn child ever. Hence, the male offspring population constituted of 246,559 (51%) mother–child pairs and the female of 233,408 (49%) pairs. The flowchart depicted in Fig. 1 shows the exclusion criteria.

### Exposures

Maternal job code during pregnancy was obtained from the OIR, coded according to the Danish version of the International Standard Classification of Occupations from 1988 (DISCO-88) and assessed on a yearly basis. Maternal job codes were linked with exposure estimates on occupational psychosocial stressors and ergonomic strain based on JEMs at the most detailed DISCO-88 level possible. The included JEMs were developed based on self-reported data from the Work Environment and Health in Denmark (WEHD) cohort survey (Madsen et al. 2018). Exposure estimates from the JEMs represent age- and sex-specific average assessments of the working condition within a group with the same job code. The predicted mean level of the occupational exposures was estimated using a generalized linear mixed model with a random intercept for job code and the estimated best linear unbiased (BLUP) estimator (Flachs et al. 2019; Madsen et al. 2018).

The JEMs on occupational psychosocial stressors were developed upon Karasek’s Job Strain Model (Karasek 1979; Madsen et al. 2018). We investigated the two dimensions of the model, i.e., quantitative demands (representing job demands) and decision authority (representing job control) separately and included the multiplicative term of the two dimensions as a covariate. This is in line with recommendations and previous research (Liu et al. 2019; Mikkelsen et al. 2018; Pape et al. 2020). For the five questions on quantitative demands and two on decision authority answers ranged on a scale from 1 to 5 and the mean score including all items of each dimension was constructed. The eight questions for the dimensions on the ergonomic JEM (sitting, walking/standing, working with the back twisted/bent, arms lifted above the shoulders, repetitive arm movements, squatting/kneeling, pushing/pulling, carrying/lifting) were answered on a scale ranging from 1 to 6 and the mean score of the sum of all items was calculated (Madsen et al. 2018). Due to the use of JEMs, scores on the exposures among the included women distributed narrowly around the mean (most pronounced for the psychosocial JEMs). To ensure contrast between low and high exposure and to avoid that results would be influenced by a large group of women with a score close to the mean, three exposure groups were constructed (*low*; *medium*; *high*).

### Outcome

Offspring asthma (*yes*; *no*) was determined by the first hospital contact due to asthma in offspring aged 0–10 years, extracted from the Danish National Patient Register (NPR) using the diagnoses of asthma (ICD-10: J45) and status asthmaticus (ICD-10: J46).

### Covariates

Potential confounding variables were identified from current literature and by Acyclic Directed Graphs (DAGs) (Greenland et al. 1999) (Figure S1): maternal asthma identified as asthma hospital contacts during the two years prior to childbirth (ICD-10: J45-J46); age at conception calculated based on the assumption that all children were born at term (day of childbirth–280 days); socioeconomic position (SEP) as the highest achieved education at conception; body mass index (BMI) retrieved from a JEM; and calendar year of child birth.

### Statistical procedures

Baseline characteristics of the study population are presented according to exposure status of occupational psychosocial stressors and ergonomic strain in Table 1. If distribution of baseline characteristics were similar among male and female offspring, proportions were shown combined for male and female offspring.

Poisson regression models were used to estimate incidence rate ratios (IRR) with corresponding 95% confidence intervals (CI) for the risk of asthma by maternal exposure to occupational psychosocial stressors and ergonomic strain separately for male and female offspring, respectively. Offspring was followed from day of birth (January 1st, 1996, earliest) and until day of the first hospital contact for asthma, emigration, death, 11-year birthday, or end of follow-up December 31st, 2018, whichever came first. Calendar year was included as a time dependent variable to account for differences in diagnostic practice and changes in occupational strain during the study period. In the Poisson regression, the assumption that rates are constant within time periods and that each event occurs independently within each time interval (Kirkwood and Sterne 2003) was tested and found satisfactorily fulfilled.

Mothers with low quantitative demands, high decision authority, and low ergonomic strain served as reference groups, and analyses were adjusted for maternal asthma, age at conception, SEP, and BMI, and calendar year. All three exposures were mutually adjusted for each other.

To explore the influence of potentially lower validity of asthma diagnoses in children below 3 years of age (Bacharier et al. 2008; Chung 2022; Martinez et al. 1995; Pedersen 2007), we performed a sensitivity analysis in which only children aged 3–10 were included, regardless of their asthma status at the age of 0–2 years.

All statistical analyses were performed in SAS 9.4 (SAS Institute Inc., Cary, NC, USA).

### Ethical considerations

The EXIMIOUS project was approved by the Knowledge Centre on Data Protection Compliance under the records of processing regarding health science research projects within the Capital Region of Denmark (Privacy: P-2020–719) and Statistics Denmark (Ref-no.: 708046).

## Results

A total of 13,201 (5%) male and 7452 (3%) female offspring in the cohort were diagnosed with asthma. Table 1 presents the baseline characteristics of the study population according to maternal level of quantitative demands, decision authority, and ergonomic strain during pregnancy for male and female offspring combined.

**Table 1:** Baseline characteristics of the full study population of 479,967 firstborn siblings born between 1996–2018 and their mothers according to maternal occupational psychosocial stressors (quantitative demands and decision authority) and ergonomic strain (combined male and female offspring)

	Total	Quantitative demands			Decision authority			Ergonomic strain		
		Low	Medium	High	Low	Medium	High	Low	Medium	High
All offspring, n [%]	479,967 [100]	283,315 [59]	157,177 [33]	39,475 [8]	36,407 [8]	317,262 [66]	126,298 [26]	183,744 [38]	229,625 [48]	66,598 [14]
Asthma, n [%]	20,653 [4]	13,463 [5]	6095 [4]	1095 [3]	1759 [5]	14,094 [4]	4800 [4]	6717 [4]	10,373 [5]	3563 [5]
Maternal age at conception, n [%]										
20–24 years	102,525 [21]	87,002 [31]	14,203 [9]	1320 [3]	13,104 [36]	75,083 [24]	14,338 [11]	17,042 [9]	48,410 [21]	37,073 [56]
25–29 years	222,032 [46]	128,993 [46]	77,367 [49]	15,672 [40]	15,739 [43]	157,166 [50]	49,127 [39]	83,629 [46]	117,415 [51]	20,998 [32]
30–34 years	117,561 [24]	51,492 [18]	50,129 [32]	15,940 [40]	6335 [17]	68,765 [22]	42,461 [34]	60,768 [33]	50,202 [22]	6591 [10]
35–44 years	37,849 [8]	15,828 [6]	15,478 [10]	6543 [16]	1229 [3]	16,248 [5]	20,378 [16]	22,305 [12]	13,598 [6]	1946 [3]
Maternal highest achieved education at conception, n [%]										
Primary school	56,342 [12]	50,131 [18]	5412 [3]	799 [2]	7901 [22]	36,363 [11]	12,078 [10]	6738 [4]	28,705 [13]	20,899 [31]
Upper secondary	207,822 [43]	157,675 [56]	43,728 [28]	6419 [16]	18,286 [50]	140,751 [44]	48,785 [39]	68,621 [38]	97,862 [43]	41,339 [62]
Vocational training	25,518 [5]	14,287 [5]	9513 [6]	1718 [4]	2075 [6]	14,985 [5]	8458 [7]	14,270 [8]	9974 [4]	1274 [2]
Bachelors	126,486 [26]	54,025 [19]	65,737 [42]	6724 [17]	3195 [9]	96,504 [30]	26,787 [21]	36,546 [20]	87,485 [38]	2455 [4]
Masters or higher	63,799 [13]	7197 [3]	32,787 [21]	23,815 [60]	4950 [14]	28,659 [9]	30,190 [24]	57,546 [31]	5599 [4]	631 [1]
Maternal Body Mass Index (JEM*), n [%]										
Low	132,447 [28]	60,986 [22]	57,900 [37]	13,561 [34]	8,431 [23]	100,092 [32]	23,924 [19]	60,457 [33]	61,612 [27]	10,378 [16]
Medium	314,925 [66]	194,814 [69]	94,980 [60]	25,131 [64]	26,207 [72]	198,694 [63]	90,024 [71]	118,505 [64]	145,330 [63]	51,090 [77]
High	32,595 [7]	27,515 [10]	4297 [3]	783 [2]	1769 [5]	18,476 [6]	12,350 [10]	4782 [3]	22,683 [10]	5130 [8]
Maternal asthma prior to childbirth, yes n [%]	1936 [1]	1227 [1]	540 [1]	169 [1]	136 [1]	1308 [1]	492 [1]	698 [1]	919 [1]	319 [1]
Calendar year at birth, n [%]										
1996–2000	100,953 [21]	60,864 [21]	35,625 [23]	4464 [11]	10,292 [28]	68,362 [22]	22,299 [18]	38,282 [21]	45,318 [20]	17,353 [26]
2001–2005	109,431 [23]	69,710 [25]	33,309 [21]	6412 [16]	8460 [23]	69,151 [22]	31,820 [25]	39,894 [22]	54,392 [24]	15,145 [23]
2006–2010	109,745 [23]	64,842 [23]	36,833 [23]	8070 [20]	7819 [21]	69,807 [22]	32,119 [25]	41,424 [23]	53,644 [23]	14,677 [22]
2011–2018	159,838 [33]	87,899 [31]	51,410 [33]	20,529 [52]	9836 [27]	109,942 [35]	40,060 [32]	64,144 [35]	76,271 [33]	19,423 [29]

*JEM Job Exposure Matrix

Among the mothers, 8% worked in occupations with high quantitative demands and low decision authority, respectively, and 14% in occupations with high ergonomic strain during pregnancy. The distribution of most baseline characteristics varied across low, medium, and high exposure for the two psychosocial stressors and ergonomic strain. Mothers in occupations with high quantitative demands were older, had longer education, and belonged to a job group with lower BMI than mothers in occupations with low quantitative demands. Mothers in occupations with low decision authority were younger and had shorter education than mothers in occupations with high decision authority. Mothers in occupations with high ergonomic strain were younger, belonged to a job group with higher BMI, and had shorter education than mothers in occupations with low ergonomic strain.

### Main analyses

In Table 2, we present the associations between maternal psychosocial stressors and ergonomic strain and the risk of asthma in male and female offspring.

**Table 2:** Crude and adjusted incidence rate ratios (IRR) and 95% confidence intervals (CI) between maternal occupational psychosocial stressors (quantitative demands and decision authority) and ergonomic strain and asthma in 497,967 male and female first-born offspring aged 0–10 years, born 1996–2018

Exposure status	No. of cases	Person years	IR per 100,000 person years	IRR (95% CI)	
				Minimally adjusted^a^	Additionally adjusted^b^
*Males (n = 246,559) [51%]*					
Quantitative demands					
Low	8575	1,168,915	733	1.00 (ref.)	1.00 (ref.)
Medium	3904	636,601	613	0.83 (0.79–0.86)	0.97 (0.92–1.01)
High	722	132,691	544	0.67 (0.62–0.72)	0.96 (0.87–1.05)
Decision authority					
High	3093	515,929	599	1.00 (ref.)	1.00 (ref.)
Medium	8980	1,267,666	708	1.17 (1.12–1.21)	1.08 (1.03–1.13)
Low	1128	154,612	729	1.23 (1.15–1.32)	1.08 (1.00–1.16)
Ergonomic strain					
Low	4269	730,926	584	1.00 (ref.)	1.00 (ref.)
Medium	6681	927,741	720	1.23 (1.19–1.28)	1.09 (1.04–1.14)
High	2251	279,539	805	1.40 (1.33–1.48)	1.09 (1.02–1.16)
*Females (n = 233,408) [49%]*					
Quantitative demands					
Low	4888	1,128,262	433	1.00 (ref.)	1.00 (ref.)
Medium	2191	615,457	356	0.81 (0.77–0.85)	0.96 (0.91–1.03)
High	373	126,881	294	0.61 (0.55–0.68)	0.91 (0.80–1.03)
Decision authority					
High	1707	494,848	345	1.00 (ref.)	1.00 (ref.)
Medium	5114	1,227,001	416	1.19 (1.13–1.26)	1.10 (1.04–1.17)
Low	631	148,751	424	1.25 (1.14–1.37)	1.06 (0.96–1.17)
Ergonomic strain					
Low	2448	704,466	347	1.00 (ref.)	1.00 (ref.)
Medium	3692	898,056	411	1.18 (1.12–1.25)	1.02 (0.95–1.08)
High	1312	268,078	489	1.43 (1.34–1.53)	1.05 (0.97–1.15)

*IR* Incidence rate, *Ref* Reference

^a^Adjusted for age of offspring

^b^Adjusted for age of offspring, maternal asthma, maternal age at conception, maternal socioeconomic position, maternal body mass index, and calendar year. Exposure variables are mutually adjusted

Asthma risk tended to be decreased in male and female offspring of mothers in occupations with medium (male: IRR: 0.83, 95% CI 0.79–0.86; female: IRR: 0.81, 95% CI 0.77–0.85) and high (male: IRR: 0.67, 95% CI 0.62–0.72; female: IRR: 0.61, 95% CI 0.55–0.68) quantitative demands in minimally adjusted analyses. However, when additionally adjusting for all confounding variables the observed associations diminished, with confidence intervals now overlapping the reference value.

Regarding decision authority, male offspring of mothers in occupations with medium (IRR: 1.08, 95% CI 1.03–1.13) or low (IRR: 1.08, 95% CI 1.00–1.16) decision authority exhibited elevated risks of asthma compared to those of mothers in occupations assigned high decision authority in the additionally adjusted analysis, where effect size was somewhat attenuated compared to the minimally adjusted analysis. Observed associations in the additionally adjusted analyses among female offspring were largely similar, but less consistent (additionally adjusted medium: IRR: 1.10, 95% CI 1.04–1.17; additionally adjusted low: IRR: 1.06, 95% CI 0.96–1.17) to the additionally adjusted analyses on male offspring. Observations on female offspring were largely attenuated from the minimally to additionally adjusted analyses.

For ergonomic strain, the additionally adjusted analyses showed that male offspring of mothers in occupations with medium (IRR: 1.09, 95% CI 1.04–1.14) or high (IRR: 1.09, 95% CI 1.02–1.16) ergonomic strain had increased risks of asthma relative to those of mothers in occupations with low ergonomic strain. In females, the additionally adjusted analyses showed an increased risk of offspring asthma (medium: IRR: 1.02, 95% CI 0.95–1.08; high: IRR: 1.05, 95% CI 0.97–1.15). However, the observed CIs were comparable to no effect on female offspring. The observations on male and female offspring were attenuated largely from the minimally adjusted analyses.

No statistically significant multiplicative interaction was observed between quantitative demands and decision authority for either sex (p > 0.05).

### Sensitivity analyses

Increasing the specificity of the asthma diagnosis, by restricting the analysis to offspring aged 3–10 years, strengthened the association between decision authority and asthma among male offspring (medium decision authority: IRR: 1.16, 95% CI 1.09–1.24; low decision authority: IRR 1.21, 95% CI 1.08–1.35) compared to the associations observed in the main analysis on male offspring aged 0–10 years (Table 3). For the remaining associations among male and female offspring, the observations were similar to those in the main analysis.

**Table 3:** Crude and adjusted incidence rate ratios (IRR) and 95% confidence intervals (CI) between maternal occupational psychosocial stressors (quantitative demands and decision authority) and ergonomic strain and asthma in 407,613 male and female first-born offspring aged 3–10 years, born 1996–2018

Exposure status	No. of cases	Person years	IR per 100,000 person years	IRR (95% CI)	
				Minimally adjusted^a^	Additionally adjusted^b^
*Males (n = 209,221) [51%]*					
Quantitative demands					
Low	3582	803,780	445	1.00 (ref.)	1.00 (ref.)
Medium	1759	431,630	407	0.91 (0.86–0.96)	0.95 (0.89–1.02)
High	325	82,415	394	0.83 (0.74–0.93)	1.00 (0.87–1.14)
Decision authority					
High	1299	351,104	370	1.00 (ref.)	1.00 (ref.)
Medium	3857	859,366	448	1.20 (1.13–1.28)	1.16 (1.09–1.24)
Low	510	107,355	475	1.29 (1.17–1.43)	1.21 (1.08–1.35)
Ergonomic strain					
Low	1898	491,676	386	1.00 (ref.)	1.00 (ref.)
Medium	2839	631,800	449	1.16 (1.10–1.23)	1.12 (1.04–1.20)
High	929	194,350	478	1.25 (1.15–1.35)	1.13 (1.02–1.25)
*Females (n = 198,392) [49%]*					
Quantitative demands					
Low	2005	767,169	261	1.00 (ref.)	1.00 (ref.)
Medium	953	413,976	230	0.87 (0.81–0.94)	0.98 (0.89–1.07)
High	165	78,541	210	0.75 (0.64–0.88)	0.99 (0.82–1.19)
Decision authority					
High	732	333,329	219	1.00 (ref.)	1.00 (ref.)
Medium	2135	823,715	259	1.17 (1.07–1.27)	1.11 (1.02–1.21)
Low	256	102,642	249	1.15 (0.99–1.32)	1.03 (0.88–1.19)
Ergonomic strain					
Low	1055	470,767	224	1.00 (ref.)	1.00 (ref.)
Medium	1546	604,815	255	1.14 (1.05–1.23)	1.03 (0.94–1.14)
High	522	184,104	283	1.27 (1.15–1.41)	1.03 (0.91–1.18)

*IR* Incidence rate, *Ref* Reference

^a^Adjusted for age of offspring

^b^Adjusted for age of offspring, maternal asthma, maternal age at conception, maternal socioeconomic position, maternal body mass index, and calendar year. Exposure variables are mutually adjusted

## Discussion

### Main findings

In this nationwide cohort study, we investigated the sex-specific associations between maternal occupational psychosocial stressors and ergonomic strain during pregnancy and the risk of offspring asthma diagnosed before 11 years of age. As hypothesized, we observed higher risks of asthma in offspring of mothers in occupations with low compared to high levels of decision authority and with high compared to low ergonomic strain during pregnancy, most pronounced for male offspring. In contrast to our hypothesis, we found no indications of lower risk of asthma in offspring of mothers in occupations with higher levels of quantitative demands.

### Main findings in the context of previous studies

The main finding of increased risk of asthma among offspring to mothers in occupations with low and medium compared to high levels of decision authority during pregnancy is partly in line with a previous Danish register-based study using the Job Strain Model. In this study, the authors observed that maternal low job control during pregnancy was associated with higher risk of early-onset asthma (before age 3 years) in the offspring (Liu et al. 2019). Previous studies from the general population lend some support that women experiencing high strain at work (high demands and low control) have elevated salivary cortisol concentrations compared to women experiencing low strain at work (low demands and high control) (Fujiwara et al. 2004; Steptoe et al. 2000), whilst others did not (Alderling et al. 2006). Increased maternal cortisol levels during pregnancy is proposed to disturb fetal development of the HPA axis with potential implications for the development of offspring asthma (Rakers et al. 2020; Wright 2008). However, as these findings are conflicting, more research is needed to elucidate the biological pathway(s) between maternal occupational psychosocial stressors during pregnancy and the development of offspring asthma.

In the minimally adjusted analyses, we observed a tendency to decreased risk of asthma due to maternal quantitative demands. However, this was diminished in the additionally adjusted analyses. The finding of no risk of asthma in offspring of mothers in occupations with increased quantitative demands in the additionally adjusted analyses is somewhat in line with previous studies. Pape et al (2020) did not observe any associations between maternal job demands and risk of asthma, while Liu et al (2019) only observed a small tendency to increased risk of early-onset asthma (before age 3 years) in offspring to mothers with high job demands, but not for late-onset asthma (Liu et al. 2019; Pape et al. 2020). These findings could reflect strong co-variation between high quantitative demands and high decision authority, called the active group in the Job Strain Model (Karasek 1979). Among mothers in occupations with high quantitative demands, 61% also had high decision authority (data not shown). Larsen et al. (2014) observed increased risk of asthma in offspring of mothers in the active group but found no association if the mother had high job demands and low job control (i.e., the high strain group) (Larsen et al. 2014). These results indicate that it is challenging to isolate the effect of maternal high job demands from the effect of maternal job control.

We are the first to study and to show a modest association between ergonomic strain during pregnancy and offspring asthma. In a meta-analysis, Cai et al (2020) found that pregnant women with heavy lifting, prolonged standing, and in a generally high physical workload had increased risk of having a low birthweight or a small-for-gestational-age child (Cai et al. 2020), both potential indicators of fetal growth restriction (Liu et al. 2014). It is well-established that low birthweight is associated with increased risk of asthma (Xu et al. 2014). Hence, our findings on ergonomic strain during pregnancy could be a result of low birthweight, which we were not able to investigate in the present study. Whether heavy ergonomic strain during pregnancy can contribute directly to the development of asthma remains to be further elucidated.

It has previously been suggested, that male offspring may be more vulnerable to environmental factors in fetal life (Chowdhury et al. 2021; Saif et al. 2014). We found no indications of difference in asthma risk due to maternal occupational psychosocial stressors. This is in line with two previous Danish studies, in which no differences between occupational psychosocial stressors on the risk of asthma between male and female offspring neither was observed (Liu et al. 2019; Pape et al. 2021). However, we observed a minor sex difference in risk of asthma due to maternal occupational ergonomic strain, in which male offspring were somewhat more vulnerable.

### Methodological considerations

The use of nationwide register-based data ensured a large sample size with high statistical power and complete follow-up for almost the entire population of Danish women working during pregnancy. The use of JEMs made it possible to assess exposure independent of self-reports and minimize the risk of reporting bias. Also, the use of JEMs allowed to us explore occupational exposures in an unselected population also including women with lower SEP. This is important as this group is less likely to participate in mother–child cohorts by self-selection (Jacobsen et al. 2010).

JEMs was used to assign occupational exposures, consequently assigning all women within a specific job the same level of occupational exposures. Differences in levels of stressors and strain is expected between employees within an occupation, and the JEM-based assignment of an average exposure to all women are prone to Berkson’s error resulting in non-differential misclassification and too narrow CIs for the estimated associations. Due to the statistical procedures performed, we cannot rule out the possibility of attenuation bias in estimates as well (Armstrong 1998). However, individual-level measurement of exposures and thus non-differential misclassification would most likely also be susceptible to such attenuation bias.

Another source of intra-job exposure variability in our study is that it lacks data from pregnant women since especially ergonomic strain may differ between pregnant and non-pregnant employees. For instance, pregnant women may consciously or subconsciously reduce their exposure to strain or opt sick leave upon pregnancy. Consequently, women identified as experiencing high-strain conditions according to the JEM may not actually encounter such strain during pregnancy, leading to a systematic misclassification and overestimation of exposure. Moreover, since sick leave is more common among pregnant women facing higher levels of strain and strenuous occupations are often low-SEP occupations, the extent of exposure misclassification becomes dependent on maternal SEP. Similarly, the prevalence of hospital-diagnosed asthma, our chosen outcome, is influenced by maternal SEP, primarily due to the differential healthcare-seeking behavior between socioeconomic groups. Especially the very small children of mothers with lower SEP are more prone to have hospital contact due to asthma (Gong et al. 2014), whereas those children of families with higher SEP are more likely to receive diagnoses through general practitioners, a factor not captured by our dataset. Consequently, the influence of maternal SEP on both our exposure assessment and outcome measurement may contribute to a systematic overestimation of the association between high strain and offspring asthma. This overestimation occurs as more asthma cases in our data are attributed to high exposure levels, where the actual exposure might be low, while simultaneously overlooking cases of higher-SEP mothers belonging to the low-strain group. Adjustment for SEP may have minimized this source of bias but could also hide associations. We used highest achieved educational level as a proxy for maternal SEP. Other proxies, e.g., income, could have been used. The information on educational level was obtained from registers and considered of high quality (Jensen and Rasmussen 2011). Educational level was chosen as it reflects certain aspects of SEP such as health-related knowledge and adoption to health behaviors (Khalatbari-Soltani et al. 2022), and thereby relevant for behavior in relation to occupational environment during pregnancy. However, educational level is closely correlated to occupation and therefore to the occupational exposure the women were assigned, and thus adjustment for SEP may cause adjustment for some of the effect of exposure as well.

The used JEMs are based on the 2012 edition of the WEHD but were applied to the period 1996–2018. We cannot preclude that misclassification on level of exposures might occur in the beginning of the cohort due to changing working conditions during the period. Further, diagnostic practice of asthma has changed during the years, potentially impacting the tendency to be diagnosed over the years. This threatens the ability of comparison over the study period. However, calendar year was included in the analyses to adjust for such changes during the follow-up period.

Residual confounding may also be of concern. Maternal asthma was identified based on hospital admission during the two years before childbirth, not redemption of prescriptions of asthma medication. Severity of asthma is a risk factor for hospitalization among adults with asthma (Eisner et al. 2001), and our prevalence of ~ 1% of maternal asthma is therefore underestimated and the analyses are inadequately adjusted for maternal asthma. BMI information was retrieved from a JEM based on general Danish population surveys (Bondo Petersen et al. 2018); and will not reflect the actual differences in BMI among the Danish working population, but will reflect overall differences between different job groups. Furthermore, the JEM was not developed on pregnant women specifically. However, as it is standard to use pre-pregnancy BMI in studies assessing maternal exposures and offspring health, the use of the JEM to assign a measure of BMI must be of limited concern in this respect.

It is challenging to diagnose asthma in infants and toddlers, as coughing and wheezing are common symptoms among small children, also without relation to asthma (Bacharier et al. 2008; Chung 2022; Martinez et al. 1995; Pedersen 2007). We, therefore, performed a sensitivity analysis with follow-up starting at 3 years of age to improve the specificity of diagnosis. This analysis revealed similar effect estimates for most associations. Thus, our results are only marginally influenced by inclusion of the very young children.

In summary, this study provided limited evidence of association between maternal occupational psychosocial stressors and ergonomic strain during pregnancy and the risk of offspring asthma. However, the lack of JEMs specifically designed for pregnant women and of adjustment for sick leave during pregnancy as well as limiting of the outcome measures to hospital-diagnosed asthma may have introduced misclassification and bias.

## Supplementary Information

Below is the link to the electronic supplementary material.

Supplementary file1 (DOCX 46 KB)

## Data Availability

The data utilized in this study cannot be made available, since the data is located at a secure server at Statistics Denmark.
