# Supplementary material for: Occupational psychosocial stressors and ergonomic strain during pregnancy and sex-specific risk of childhood asthma
Source: Int Arch Occup Environ Health. 2024 Dec 4;98(1):13–23. doi: 10.1007/s00420-024-02107-6 (PMC11807018; doi:10.1007/s00420-024-02107-6)
Supplement: Supplementary file 1 — Supplementary file1 (DOCX 46 KB) [file 420_2024_2107_MOESM1_ESM.docx]

**Figure S1.** Directed Acyclic Graph (DAG) depicting the assumed causal relationship between maternal occupational psychosocial stressors and ergonomic strain and asthma in the offspring.


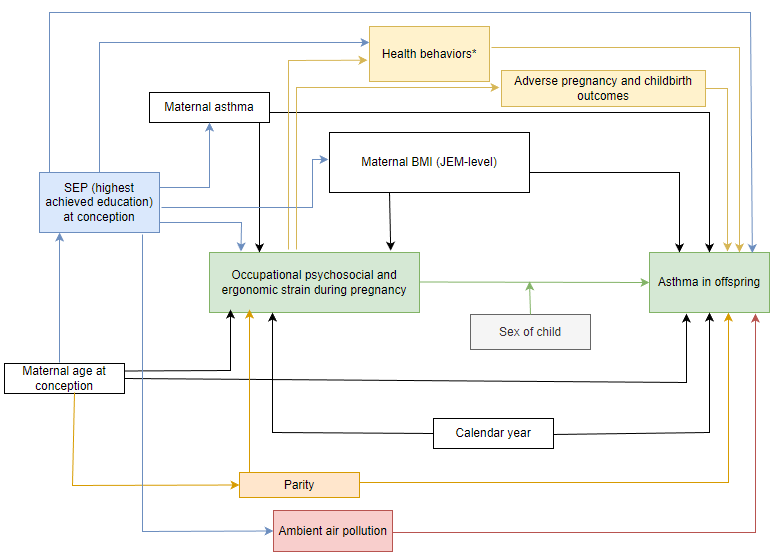


Green boxes represent the exposure and outcome, white boxes are potential confounding variables. The yellow box represents unmeasured health behaviors. The blue box illustrates a variable only adjusted for in the additionally adjusted model. The orange box presents a variable only adjusted for in a supplementary analysis on random sibling. The grey box presents the potential effect modification of sex on the association between exposure and outcome. The red box presents an unmeasured potential confounding variable.

*Health behaviors include smoking, alcohol intake, and leisure time physical activity.
